# The IRAK Homolog Pelle Is the Functional Counterpart of IκB Kinase in the *Drosophila* Toll Pathway

**DOI:** 10.1371/journal.pone.0075150

**Published:** 2013-09-23

**Authors:** Jessica Daigneault, Liv Klemetsaune, Steven A. Wasserman

**Affiliations:** Section of Cell and Developmental Biology, University of California San Diego, La Jolla, California, United States of America; University of Massachusetts Medical School, United States of America

## Abstract

Toll receptors transduce signals that activate Rel-family transcription factors, such as NF-κB, by directing proteolytic degradation of inhibitor proteins. In mammals, the IκB Kinase (IKK) phosphorylates the inhibitor IκBα. A βTrCP protein binds to phosphorylated IκBα, triggering ubiquitination and proteasome mediated degradation. In *Drosophila*, Toll signaling directs Cactus degradation via a sequence motif that is highly similar to that in IκBα, but without involvement of IKK. Here we show that Pelle, the homolog of a mammalian regulator of IKK, acts as a Cactus kinase. We further find that the fly βTrCP protein Slimb is required in cultured cells to mediate Cactus degradation. These findings enable us for the first time to trace an uninterrupted pathway from the cell surface to the nucleus for *Drosophila* Toll signaling.

## Introduction

Toll and Toll-like receptors (TLRs) govern the nuclear localization and activity of NF-κB and other Rel-related transcription factors via an evolutionarily conserved signal transduction pathway [1], [2], [3]. Prior to Toll signaling, Rel proteins are held in the cytoplasm by a tightly bound inhibitor that belongs to the IκB family. Activated Toll receptors relay signals that trigger IκB protein degradation, freeing the Rel proteins to translocate into nuclei, bind DNA, and regulate gene expression.

In the fruit fly *Drosophila melanogaster*, Toll signaling functions in both development and immunity [4], [5]. Toll first acts in the syncytial embryo, where spatially graded signaling establishes the dorsoventral axis. In larvae and adults, Toll mediates the humoral immune response to fungi and Gram (+) bacteria. Embryonic axis formation requires the Rel protein Dorsal, whereas innate immune responses involve either Dorsal or the Dorsal-related immune factor (Dif), another Rel protein [6]. Cactus, a fly IκB protein, is the inhibitor for both Dif and Dorsal. The adaptor proteins MyD88 and Tube, as well as the protein kinase Pelle, transduce signals from Toll to Cactus. These three signal-relay proteins each contain a death domain, a protein-protein interaction motif that mediates formation of a submembranous Toll signaling complex [7], [8].

Like *Drosophila* Toll, most mammalian TLRs signal via three death domain proteins. Indeed, mammalian MyD88, IRAK4 and IRAK1 are the counterparts of fly MyD88, Tube, and Pelle, respectively [9], [10]. However, additional factors link the death domain complex to IκB in mammalian innate immune signaling. In particular, signaling by the IRAK 1,2, and 4 proteins requires the adaptor TRAF6, the TAB proteins, the protein kinase TAK1, and the IκB Kinase (IKK) complex [1], [11]. IKK-mediated phosphorylation of IκBα at two sites, Ser32 and Ser36, triggers ubiquitination, leading to proteasome mediated IκBα degradation [12], [13].

The Toll responsive sites in IκBα and Cactus share substantial sequence similarity [12], [14], [15]. In fact, the signal-responsive domain of IκBα can functionally substitute for the corresponding region of the Cactus protein [16]. Surprisingly, the *Drosophila* IKK does not function in the fly Toll pathway [17], [18], [19].

The fact that Toll-directed Cactus phosphorylation is IKK independent leads to two important questions. First, why are the signal responsive sites conserved when the kinase is not? Second, what kinase phosphorylates these sites in response to Toll activation? Here we address these two questions.

## Results

### *Drosophila* β-TrCP can Mediate Cactus Degradation

**Figure 1:**
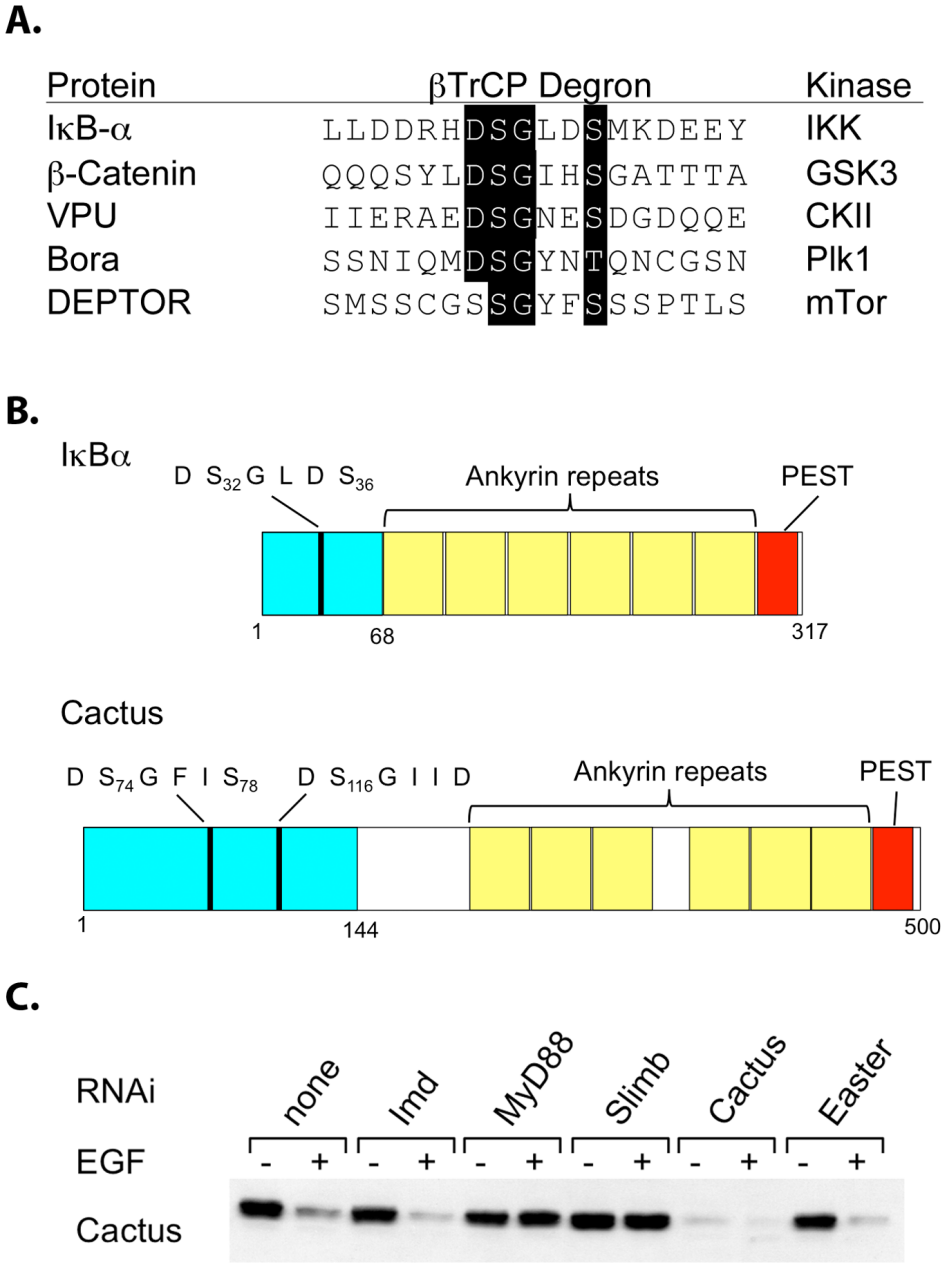
Role of Slimb βTrCP in Toll-directed Cactus degradation. A. The βTrCP recognition sites and protein kinases of well-characterized degrons (see [21], and references therein). B. Schematic drawing of IκB and Cactus proteins (adapted from [16]). Teal colored regions represent signal-responsive domains. C. Anti-Cactus immunoblot of EGFR-Toll expressing S2 cells treated with indicated dsRNA’s and activated with EGF.

To explain the sequence similarity of the signal responsive sites in IκBα and Cactus, it has been proposed that Cactus, like IκBα, is targeted for degradation by a β-TrCP protein acting as the substrate recognition subunit for an SCF E3 ubiquitin ligase [16], [20]. In mammals, the F-box/β-TrCP protein of the SCF (Skp1/Cullin/F-Box) complex binds specifically to the phosphorylated form of the IκBα motif DS_32_GLDS_36_. This recognition initiates ubiquitination, generating the signal for proteasomal recognition and proteolysis [1], [21], [22]. The recognition site for β-TrCP is thus a degron that initiates protein degradation in response to phosphorylation. Characterization of the degrons of a number of β-TrCP targets has demonstrated that they are highly similar in sequence, with a consensus sequence of DSGxxS, but are regulated by a diverse set of protein kinases (Figure 1A).

As shown in Figure 1B, both of the signal-responsive sites in Cactus exhibit similarity to β-TrCP sites, with the sequence from residues 73–78 forming a perfect match to the consensus recognition site. In flies, however, evidence for a role of β-TrCP protein in Cactus degradation is somewhat contradictory. The *slimb* locus is the sole β-TrCP gene in the *Drosophila* genome. Whereas a loss-of-function mutation in *slimb* blocks the Toll-dependent transcription of the patterning genes *snail* and *twist* in embryos, Toll-dependent transcription of the antifungal gene *drosomycin* is unaffected in adults [20], [23]. Furthermore, ectopic expression of the viral β-TrCP inhibitor VPU in flies reduces but does not eliminate Pelle-mediated degradation of Cactus [20].

To investigate further the role of the Slimb β-TrCP in Cactus degradation, we used S2 cells transfected with a construct fusing the extracellular domain of EGFR to the transmembrane and intracellular domains of Toll. We have previously demonstrated that this chimeric receptor renders the Toll pathway responsive to EGF, directing Cactus degradation that is detectable within one minute of EGF addition [24]. To assay the requirement for Slimb in Toll signaling, we used EGF activation of EGFR-Toll in combination with RNA interference (RNAi). The results were unequivocal. As shown in Figure 1C, RNAi against *slimb* abrogated Toll-directed degradation of Cactus. Indeed, eliminating *slimb* function had the same effect as did RNAi against *MyD88*, an essential component of the fly Toll pathway. In contrast, RNAi against *easter*, a gene that acts upstream of Toll, or against *Imd*, a gene essential to the IKK-dependent immune pathway, was without consequence. We conclude that Toll-directed Cactus degradation in these cells requires the β-TrCP protein Slimb.

### Pelle Phosphorylates Signal-dependent Sites in Cactus

Given that IKK does not mediate Toll signaling, what kinase phosphorylates Cactus in response to Toll activation? A logical candidate is Pelle. The protein kinase activity of Pelle is strictly required for Toll signaling and forced expression of Pelle drives Cactus degradation [15], [25], [26], [27]. Furthermore, Pelle undergoes autophosphorylation, autoactivation, and colocalization with Tube, events that occur concomitant with signaling [28], [29], [30]. There is also the fact that forward genetic screens and genome-wide RNAi screens have failed to identify any other protein kinase required for Cactus degradation [31], [32], [33], [34]. We therefore set out to assay Pelle as a Cactus kinase.

We began by expressing recombinant forms of Cactus and Pelle in *Escherichia coli*. We expressed full-length Cactus (500 aa), isolated the protein from inclusion bodies, and carried out solubilization and renaturation by the method of Nüsslein-Volhard and colleagues [35]. Expressing Pelle in *E. coli* proved more problematic, due to the frequent appearance of mutations that blocked Pelle production or inactivated the kinase domain. To circumvent the toxicity of an active Pelle kinase in bacteria, we adopted the strategy of co-expressing an antagonizing protein phosphatase [36], [37], [38]. For this purpose we chose λ protein phosphatase, which contains just 221 residues, is readily expressed in bacteria, and has very broad substrate specificity [39], [40].

**Figure 2:**
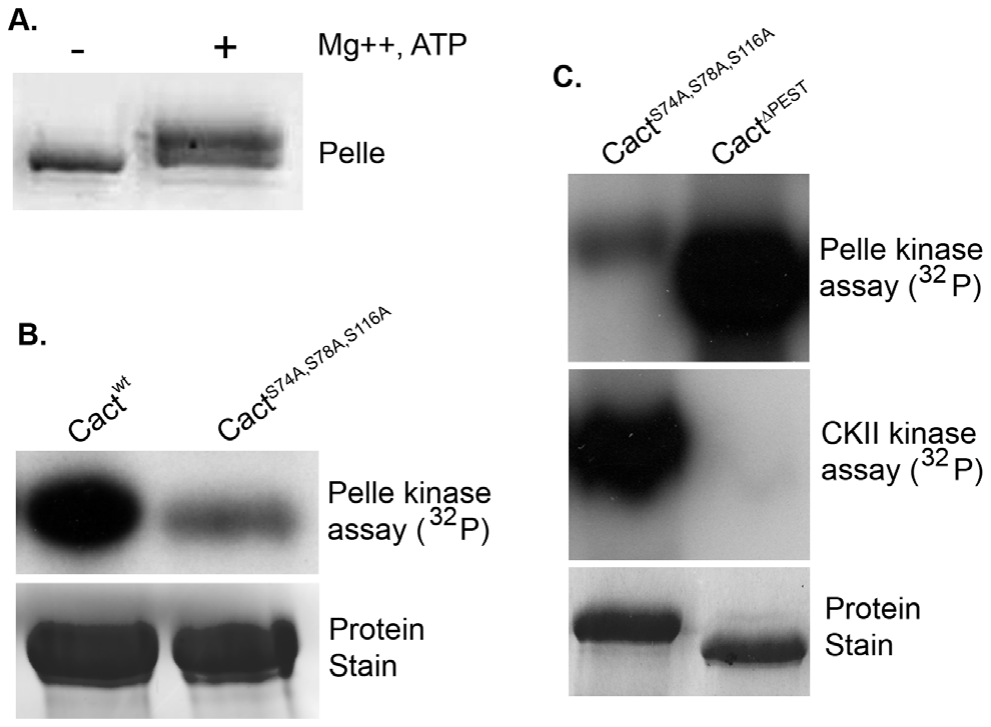
Pelle-mediated Cactus phosphorylation exhibits specificity for Toll-responsive sites. A. Autophosphorylation of recombinant GST-Pelle, stained with Coomassie brilliant blue. B. (Top) Autoradiogram of Cactus isoforms phosphorylated with GST-Pelle in the presence of [**γ**-^32^P]-ATP. (Bottom) SDS-PAGE of unlabeled substrates. C. Autoradiograms of Cactus isoforms radiolabelled with GST-Pelle or Casein Kinase II (CKII) in the presence of [**γ**-^32^P]-ATP.

To ensure coordinated expression of Pelle and the λ phosphatase, we constructed a cistron consisting of a GST-Pelle fusion gene immediately 5′ to the phosphatase gene. We induced expression in *E. coli* and used affinity chromatography to purify the Pelle fusion protein away from the λ phosphatase and other proteins. Upon incubation at high concentration with Mg^2+^ and ATP, recombinant Pelle protein underwent autoactivation and autophosphorylation, as evident in a slight decrease in electrophoretic mobility (Figure 2a). Pelle from Drosophila tissues similarly undergoes autoactivation and autophosphorylation following phosphatase treatment [30].

With an active Pelle preparation in hand, we set out to investigate Cactus as a potential substrate. We prepared two His_6_-Cactus isoforms, the wild-type Cact^wt^, and the triple mutant Cact^S74A,S78A,S116A^. In the latter isoform, the serine-to-alanine substitutions at positions 74, 78, and 116 (see Figure 1B), are sufficient to block Toll-dependent degradation of Cactus *in vivo* [14], [15], [16]. We incubated Pelle with the Cactus proteins in the presence of [**γ**-^32^P]-ATP and assayed phosphorylation by gel electrophoresis and autoradiography. We observed robust phosphorylation of wild-type Cactus, but dramatically reduced phosphorylation of the triple mutant (Figure 2B). Indeed, although the residues altered in this mutant represent only 6% of the serines in Cactus, mutating these three sites consistently reduced phosphorylation by 75–80%.

Next, we compared the site specificity of Pelle with that of Casein Kinase II (CKII). We have previously demonstrated that CKII phosphorylates target residues in the carboxyl-terminal PEST domain, mediating Toll-independent destabilization of Cactus [41]. To compare the activity of the two kinases toward Cactus, we used as substrate Cact^S74A,S78A,S116A^, in which the signal-dependent sites are mutated, and Cact^ΔPEST^, in which the signal-independent sites are deleted. As shown in Figure 2C, Pelle and CKII exhibited reciprocal specificity. Under conditions where Pelle had only minor activity toward substrate Cact^S74A,S78A,S116A^, CKII mediated extensive radiolabeling of this protein. Similarly, Pelle had robust activity toward Cact^ΔPEST^, whereas CKII catalyzed phosphorylation of this substrate was virtually undetectable. We conclude that Pelle acts as a Cactus kinase and preferentially phosphorylates Cactus at the serines required for signal responsiveness.

### Pelle Phosphorylates Signal-responsive Sites in IκBα

Stein and colleagues have shown that *Drosophila* Toll signaling can target the signal dependent sites of IκBα as well as Cactus [16]. We therefore predicted that Pelle should exhibit *in vitro* activity toward the two serines in the IκBα degron, GS_32_GLDS_36_. To test this hypothesis we turned to a chimeric substrate, IκB-CactΔ144, in which the signal-responsive region (residues 1–68) of IκBα replaces the corresponding region (residues 1–144) of Cactus (see teal colored regions in Figure 1B). This chimera not only functionally substitutes for Cactus in fly embryos, but also strictly requires residues Ser32 and Ser36 for this activity [16].

**Figure 3:**
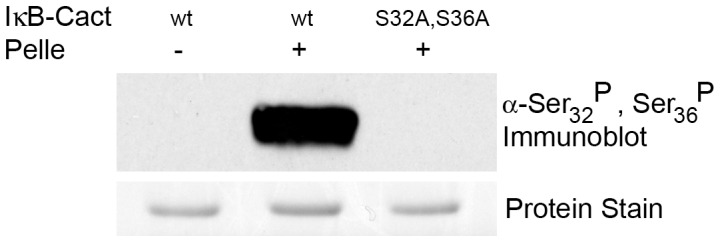
Pelle phosphorylates the IκBα degron. Isoforms of the IκB-CactΔ144 chimera were incubated with or without recombinant Pelle and the reaction products immunoblotted with an antiserum specific for the phosphorylated forms of serines 32 and 36.

We expressed and purified IκB-CactΔ144 from bacteria. We then assayed Pelle activity using an anti-IκBα serum that is specific for the IκBα phosphorylated at both Ser32 and Ser36 in the degron. As shown in Figure 3, the phospho-specific antiserum detects a strong, Pelle-dependent signal, confirming that, as predicted, Pelle directly modifies the IκBα degron, GS_32_GLDS_36_.

## Discussion

### Phosphoregulation of Cactus Stability

Studies in fly embryos have identified two processes, one signal independent and one signal dependent, that regulate Cactus stability [42]. The kinase responsible for each activity is now known. We previously used an in-gel kinase assay to purify the kinase mediating signal independent degradation [41]. We identified this kinase as Casein Kinase II (CKII) and showed that it destabilized Cactus by modifying consensus target residues in the carboxyl-terminal PEST domain. Mammalian CKII similarly modifies and destabilizes IκBα [43]. Here, we have shown that Pelle phosphorylates the signal-regulated sites in the amino-terminal domain of Cactus. Work from our lab and others has also shown that Pelle protein kinase activity is required downstream of Tube to direct Cactus degradation in response to Toll activation. Pelle thus has the *in vivo* and *in vitro* properties expected of the Cactus kinase.

Functionally, Pelle-catalyzed phosphorylation of Cactus is analogous to that of the mammalian IKK complex acting on IκBα. Different kinases mediate phosphorylation, but at highly similar sites. This site conservation has been suggested to reflect a need for SCF mediated recognition, a hypothesis supported by our finding that the Slimb β-TrCP is required in S2 cells for Cactus degradation. We note, however, that the Slimb β-TrCP may not mediate Cactus degradation in all cells and at all stages, since Lemaitre and colleagues have provided evidence that Slimb is not the sole mediator of Cactus degradation in adult flies [20]. Involvement of an additional F box protein could explain why the Cactus of flies and other invertebrates typically contains two degrons, with the more carboxyl-terminal motif deviating slightly but consistently from the β-TrCP consensus (Figure 1 and unpublished data).

### Pelle as the *Drosophila* Toll Pathway Kinase

The kinase activity of Pelle appears to fulfill multiple roles in Toll signaling. We have shown here that Pelle modifies Cactus. In addition, we and others have shown previously that Pelle phosphorylates Tube *in vitro* [27], [28]. This activity may play an *in vivo* role in terminating signal transduction, since Pelle activity as a protein kinase mediates feedback regulation on Tube localization [27]. In addition, it is known that Dorsal undergoes signal dependent phosphorylation and can respond to Toll signaling in the absence of Cactus [44], [45], [46], [47]. We speculate that Pelle is also responsible for modification of Dorsal *in vivo*.

**Figure 4:**
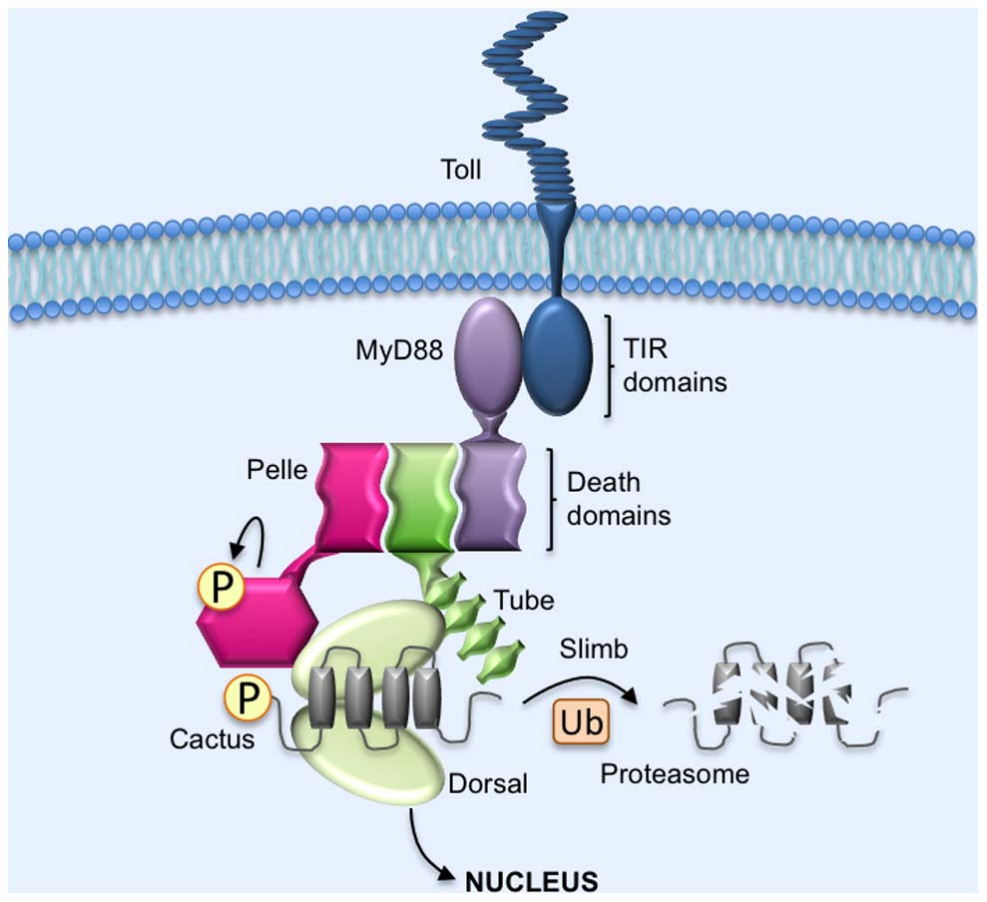
Model for Toll signaling in *Drosophila* embryos. Upon binding activated (processed) Spätzle, Toll dimerizes. The resulting conformational change allows the Toll TIR domain to bind to the TIR domain of the adaptor protein MyD88. (For simplicity, interactions and downstream events are shown for only one monomer of a dimeric Toll receptor). MyD88 brings along with it the adaptor protein Tube, an association mediated by a death domain in each protein. The bivalent Tube death domain then binds the death domain of Pelle, recruiting the inactive protein kinase into the signaling complex. Within the complex, Pelle undergoes autoactivation via autophosphorylation. Pelle then interacts with Dorsal-bound Cactus, an interaction enhanced by the binding of Dorsal to both Tube and Pelle. Pelle phosphorylates Cactus, triggering recognition by the Slimb βTrCP and subsequent ubiquitination and proteasome mediated degradation. Pelle likely also phosphorylates Dorsal, enhancing its gene regulatory activity upon release from Cactus, and Tube, initiating negative feedback regulation on Toll signaling.

By themselves, Pelle and Cactus do not appear to stably interact [28], [48]. However, Pelle binds to Tube; Tube and Pelle both bind to Dorsal; and Dorsal binds to Cactus [28], [45], [46], [48], [49], [50], [51]. Taking all of these interactions into consideration, we can now generate an illustration of the overall pathway in *Drosophila* embryos for signaling by activated Toll receptors (Figure 4).

The protein-protein interactions shown in Figure 4 likely increase the efficiency or specificity of Pelle activity when Dorsal is the Toll target. They may, for example, provide steric constraints on the interaction of Pelle and Cactus that enhance site preference. They might also explain why a Cactus isoform lacking the destabilizing effect of the PEST domain requires the presence of Dorsal for efficient response to Toll signaling [16]. However, the same interactions may not be important when Dif is the target, since Tube fails to bind Dif under conditions in which it stably binds to Dorsal [9]. Why this difference? One possibility is that the establishment of a nuclear concentration gradient of Dorsal across the embryo requires much more tightly regulated protein localization and diffusion than does the nuclear import of Dif in response to immune challenge.

Although recruitment of Toll components into a signaling complex likely influences Pelle activity, several lines of evidence reveal that such a complex is not strictly required for the action of Pelle on Cactus. First, deleting the region of Tube that mediates binding to Dorsal has a relatively mild effect on Toll signaling [48], [50], [52]. Second, expression of a fusion construct that targets Pelle to the plasma membrane can effect signaling in the absence of Tube [28], [53]. Third, activation of Toll in embryos by injection of the Toll ligand Spätzle can trigger a detectable level of Cactus degradation in the absence of Dorsal [42]. Consistent with these results, we find that Pelle preferentially phosphorylates signal responsive sites in Cactus *in vitro* in the absence of any other Toll pathway proteins.

Given that mammals require eight proteins in the portion of the Toll pathway that in flies is occupied by Pelle, it is appealing to consider the *Drosophila* pathway a model of simplicity. We note, however, that the history of Toll studies in flies and mammals is full of unexpected parallels as well as minor differences of major significance. Having offered the model shown in Figure 4, we will leave judgments regarding simplicity or complexity to others.

## Materials and Methods

### Molecular Biology

The GST Pelle-λ protein phosphatase (PPase) operon was generated by subcloning the λ-PPase gene from a pT7 vector (gift of Jack Dixon) into the Not I site of a pGEX 4T-1 vector (Amersham), and then inserting the Pelle coding sequence as an in-frame fusion with GST using EcoRI and XhoI restriction sites. The result was a 27 bp separation between coding regions of GST-Pelle and λ-PPase, with a Shine-Delgarno sequence 8 bases upstream of the phosphatase translational start site. For Cactus, PCR amplification was used to introduced an amino-terminal His_6_ tag, with the tagged construct being inserted into the BamHI and EcoRI restriction sites in the pRSET-A vector (Invitrogen). To generate the IκBα chimera, IκBα (a gift from Michael Karin) was fused with cactusΔPEST and ligated into the pRSET-B vector (Invitrogen) in frame with the polyhistidine tag. All point mutations were generated by PCR SOEing [54].

### S2 Cell Studies

S2 cells stably transfected with EGFR-Toll were treated with dsRNA and with mouse EGF, as described [24]. After 20 min, cells were harvested and 15 µg samples resolved on 8% SDS-PAGE and immunoblotted onto PVDF. Cactus was detected with a rabbit polyclonal antiserum (1∶10,000), described previously [15].

### Expression and Purification of Pelle

BL21 cells were transformed with Pelle-λPPase construct and protein expression was induced as described [39]. BL21 cells transformed with the Pelle-λPPase construct were grown for 5 hours at 28°C following IPTG induction. Cells from a 500 ml culture were pelleted and resuspended in 40 ml buffer containing 50 mM Tris HCl, pH 7.5; 2 mM EGTA; 0.5 M NaCl; and a protease inhibitor cocktail tablet (Roche). Cells were frozen and thawed; incubated with lysozyme, RNase, and DNase I in the presence of MgCl; and then lysed by three cycles of freezing (liquid nitrogen) and thawing (37°C). The lysate was spun at 25,000 g for 20 min at 4°C and the supernatant was decanted and incubated with 2 ml 50% glutathione Sepharose 4B resin slurry (GE Healthcare) in PBS for 2 hours at 4°C with gentle rocking. The beads were recovered by centrifugation at 500 g for 3 min at 4°C and the resin was washed three times in 10 ml PBS. GST-Pelle was eluted by adding 1 ml of elution buffer (10 mM glutathione, 50 mM Tris HCl, pH 8) and incubated for 1 hr at 4°C with gentle rocking.

### Expression and Purification of His_6_-cactus and Chimera

Wild-type and mutant forms of Cactus and the IκB-CactΔ144 chimera were each expressed with an amino-terminal His_6_ tag in the pRSET vector in BL21 cells. Cactus proteins were renatured according to the protocol of Nüsslein-Volhard and colleagues [35]. Wild-type and mutant forms of Cactus and the IκB-CactΔ144 chimera were each expressed with an amino-terminal His_6_ tag in the pRSET vector in BL21 cells. Expression was induced with 1 mM IPTG at 37°C for 3 hours. The lysate from a 500 ml culture was prepared by freeze/thaw cycles as described above and spun at 9000 g for 20 min. The pellet was resuspended in 20 ml buffer (20 mM Tris HCl, pH 8, 100 mM NaCl, 2 mM DTT, 2 mM EDTA, 1% Triton-X 100, ½×complete protease inhibitor tablet (Roche), stirred at 4°C for 1 hour, spun at 9000 g for 20 min, and resuspended in the same buffer without Triton-X 100. After incubation with stirring at 4°C for 1 hr, the preparation was pelleted and then resuspended to 10 mg/ml protein in urea buffer (20 mM Tris HCl, pH 8, 10 mM DTT, 1 mM PMSF, 8M urea) prior to renaturation.

### Radiolabeling Assay of Pelle

GST-pelle (10 µg) was pre-incubated for 30 min at 30°C in 1×kinase buffer (10 µM ATP, 10 mM MgCl_2_, 50 mM β-glycerophosphate, 25mM HEPES, pH 6.5) to allow activation by autophosphorylation. The activated Pelle (0.5 μg) was then incubated with recombinant His_6_-Cactus (1.6 μg) in a volume of 40 μl in the presence of [**γ** -^32^P]-ATP (13.3 μCurie, Perkin Elmer) in 1×kinase buffer. Following reaction for 5 min at room temperature, samples were mixed with 10 μl 5×SDS loading sample buffer, boiled, and loaded (30 µl) on an 8% SDS protein gel. The gel was fixed twice for 30 min in fixing solution (50% methanol, 10% acetic acid) on a shaker, before being dried in a gel dryer for 1 hr. The gel was then exposed to a film and developed. Quantitation was performed with ImageJ software.

### Immunoblot Assay of Pelle

Reactions were carried out as described above, except the kinase reactions were carried out with 11.2 μg of the chimera for 5 min at 30°C without radiolabel. Immunoblotting of the proteins transferred to a nitrocellulose membrane used rabbit anti-IκBα [pSpS^32/36^] phosphospecific antibody (Invitrogen, 1∶10,000) and α-rabbit secondary antibody (Sigma, 1∶5,000), followed by detection using the Western Lightning-ECL chemiluminescence substrate (Perkin Elmer).

## References

[1] Karin M, Ben-Neriah Y (2000) Phosphorylation meets ubiquitination: the control of NF-[kappa]B activity. Annu Rev Immunol 18: 621–663. 10837071 10.1146/annurev.immunol.18.1.621

[2] Silverman N, Maniatis T (2001) NF-kappaB signaling pathways in mammalian and insect innate immunity. Genes Dev 15: 2321–2342. 11562344 10.1101/gad.909001

[3] Akira S (2006) TLR signaling. Curr Top Microbiol Immunol 311: 1–16. 17048703 10.1007/3-540-32636-7_1

[4] Lemaitre B, Hoffmann J (2007) The host defense of Drosophila melanogaster. Annual review of immunology 25: 697–743. 10.1146/annurev.immunol.25.022106.141615 17201680

[5] Lindsay SA, Wasserman SA (2013) Conventional and non-conventional Drosophila Toll signaling Developmental & Comparative Immunology: in press. 10.1016/j.dci.2013.04.011 PMC3787077 23632253

[6] Ip YT, Reach M, Engstrom Y, Kadalayil L, Cai H, et al (1993) Dif, a dorsal-related gene that mediates an immune response in Drosophila. Cell 75: 753–763. 8242747 10.1016/0092-8674(93)90495-c

[7] Sun H, Bristow BN, Qu G, Wasserman SA (2002) A heterotrimeric death domain complex in Toll signaling. Proc Natl Acad Sci U S A 99: 12871–12876. 12351681 10.1073/pnas.202396399 PMC130552

[8] Moncrieffe MC, Grossmann JG, Gay NJ (2008) Assembly of oligomeric death domain complexes during Toll receptor signaling. The Journal of Biological Chemistry 283: 33447–33454. 18829464 10.1074/jbc.M805427200 PMC2662270

[9] Towb P, Sun H, Wasserman SA (2009) Tube Is an IRAK-4 homolog in a Toll pathway adapted for development and immunity. Journal of Innate Immunity 1: 309–321. 19498957 10.1159/000200773 PMC2690232

[10] Lin SC, Lo YC, Wu H (2010) Helical assembly in the MyD88-IRAK4-IRAK2 complex in TLR/IL-1R signalling. Nature 465: 885–890. 20485341 10.1038/nature09121 PMC2888693

[11] Kawai T, Akira S (2006) TLR signaling. Cell Death Differ 13: 816–825. 16410796 10.1038/sj.cdd.4401850

[12] Brown K, Gerstberger S, Carlson L, Franzoso G, Siebenlist U (1995) Control of IkB-a proteolysis by site-specific, signal-induced phosphorylation. Science 267: 1485–1488. 7878466 10.1126/science.7878466

[13] Chen ZJ, Parent L, Maniatis T (1996) Site-specific phosphorylation of IkappaBalpha by a novel ubiquitination-dependent protein kinase activity. Cell 84: 853–862. 8601309 10.1016/s0092-8674(00)81064-8

[14] Bergmann A, Stein D, Geisler R, Hagenmaier S, Schmid B, et al (1996) A gradient of cytoplasmic Cactus degradation establishes the nuclear localization gradient of the *dorsal* morphogen in *Drosophila* . Mechanisms of Development 60: 109–123. 9025065 10.1016/s0925-4773(96)00607-7

[15] Reach M, Galindo RL, Towb P, Allen JL, Karin M, et al (1996) A gradient of cactus protein degradation establishes dorsoventral polarity in the Drosophila embryo. Developmental Biology 180: 353–364. 8948598 10.1006/dbio.1996.0308

[16] Fernandez NQ, Grosshans J, Goltz JS, Stein D (2001) Separable and redundant regulatory determinants in Cactus mediate its dorsal group dependent degradation. Development 128: 2963–2974. 11532919 10.1242/dev.128.15.2963

[17] Silverman N, Zhou R, Stoven S, Pandey N, Hultmark D, et al (2000) A Drosophila IkappaB kinase complex required for Relish cleavage and antibacterial immunity. Genes Dev 14: 2461–2471. 11018014 10.1101/gad.817800 PMC316979

[18] Rutschmann S, Jung AC, Zhou R, Silverman N, Hoffmann JA, et al (2000) Role of Drosophila IKK gamma in a toll-independent antibacterial immune response. Nat Immunol 1: 342–347. 11017107 10.1038/79801

[19] Lu Y, Wu LP, Anderson KV (2001) The antibacterial arm of the drosophila innate immune response requires an IkappaB kinase. Genes Dev 15: 104–110. 11156609 10.1101/gad.856901 PMC312606

[20] Leulier F, Marchal C, Miletich I, Limbourg-Bouchon B, Benarous R, et al (2003) Directed expression of the HIV-1 accessory protein Vpu in Drosophila fat-body cells inhibits Toll-dependent immune responses. EMBO reports 4: 976–981. 12973300 10.1038/sj.embor.embor936 PMC1326394

[21] Skaar JR, Pagan JK, Pagano M (2013) Mechanisms and function of substrate recruitment by F-box proteins. Nature reviews Molecular cell biology 14: 369–381. 23657496 10.1038/nrm3582 PMC3827686

[22] Kanarek N, Ben-Neriah Y (2012) Regulation of NF-kappaB by ubiquitination and degradation of the IkappaBs. Immunological reviews 246: 77–94. 22435548 10.1111/j.1600-065X.2012.01098.x

[23] Spencer E, Jiang J, Chen ZJ (1999) Signal-induced ubiquitination of IkBa by the F-box protein Slimb/b-TrCP. Genes and Development 13: 284–294. 9990853 10.1101/gad.13.3.284 PMC316434

[24] Sun H, Towb P, Chiem DN, Foster BA, Wasserman SA (2004) Regulated assembly of the Toll signaling complex drives Drosophila dorsoventral patterning. The EMBO journal 23: 100–110. 14685264 10.1038/sj.emboj.7600033 PMC1271671

[25] Shelton CA, Wasserman SA (1993) pelle encodes a protein kinase required to establish dorsoventral polarity in the *Drosophila* embryo. Cell 72: 515–525. 8440018 10.1016/0092-8674(93)90071-w

[26] Hecht PM, Anderson KV (1993) Genetic characterization of tube and pelle, genes required for signaling between Toll and dorsal in the specification of the dorsal- ventral pattern of the Drosophila embryo. Genetics 135: 405–417. 8244004 10.1093/genetics/135.2.405 PMC1205645

[27] Towb P, Bergmann A, Wasserman SA (2001) The protein kinase Pelle mediates feedback regulation in the Drosophila Toll signaling pathway. Development 128: 4729–4736. 11731453 10.1242/dev.128.23.4729

[28] Grosshans J, Bergmann A, Haffter P, Nusslein-Volhard C (1994) Activation of the kinase Pelle by Tube in the dorsoventral signal transduction pathway of Drosophila embryo. Nature 372: 563–566. 7527496 10.1038/372563a0

[29] Towb P, Galindo RL, Wasserman SA (1998) Recruitment of Tube and Pelle to signaling sites at the surface of the Drosophila embryo. Development 125: 2443–2450. 9609827 10.1242/dev.125.13.2443

[30] Shen B, Manley JL (2002) Pelle kinase is activated by autophosphorylation during Toll signaling in Drosophila. Development 129: 1925–1933. 11934858 10.1242/dev.129.8.1925

[31] Brennan CA, Anderson KV (2004) Drosophila: the genetics of innate immune recognition and response. Annu Rev Immunol 22: 457–483. 15032585 10.1146/annurev.immunol.22.012703.104626

[32] Huang HR, Chen ZJ, Kunes S, Chang GD, Maniatis T (2010) Endocytic pathway is required for Drosophila Toll innate immune signaling. Proc Natl Acad Sci U S A 107: 8322–8327. 20404143 10.1073/pnas.1004031107 PMC2889516

[33] Kuttenkeuler D, Pelte N, Ragab A, Gesellchen V, Schneider L, et al (2010) A large-scale RNAi screen identifies Deaf1 as a regulator of innate immune responses in Drosophila. J Innate Immun 2: 181–194. 20375635 10.1159/000248649

[34] Valanne S, Myllymaki H, Kallio J, Schmid MR, Kleino A, et al (2010) Genome-wide RNA interference in Drosophila cells identifies G protein-coupled receptor kinase 2 as a conserved regulator of NF-kappaB signaling. J Immunol 184: 6188–6198. 20421637 10.4049/jimmunol.1000261

[35] Geisler R, Bergmann A, Hiromi Y, Nüsslein-Volhard C (1992) Cactus, a gene involved in dorsoventral pattern formation of Drosophila, is related to the IkB gene family of vertebrates. Cell 71: 613–621. 1423618 10.1016/0092-8674(92)90595-4

[36] Weijland A, Neubauer G, Courtneidge SA, Mann M, Wierenga RK, et al (1996) The purification and characterization of the catalytic domain of Src expressed in Schizosaccharomyces pombe. Comparison of unphosphorylated and tyrosine phosphorylated species. European journal of biochemistry/FEBS 240: 756–764. 10.1111/j.1432-1033.1996.0756h.x 8856081

[37] Seeliger MA, Young M, Henderson MN, Pellicena P, King DS, et al (2005) High yield bacterial expression of active c-Abl and c-Src tyrosine kinases. Protein science : a publication of the Protein Society 14: 3135–3139. 16260764 10.1110/ps.051750905 PMC2253236

[38] Wang YH, Ayrapetov MK, Lin X, Sun G (2006) A new strategy to produce active human Src from bacteria for biochemical study of its regulation. Biochemical and Biophysical Research Communications 346: 606–611. 16765913 10.1016/j.bbrc.2006.05.180

[39] Zhuo S, Clemens JC, Hakes DJ, Barford D, Dixon JE (1993) Expression, purification, crystallization, and biochemical characterization of a recombinant protein phosphatase. The Journal of biological chemistry 268: 17754–17761. 8394350

[40] Elling RA, Tangonan BT, Penny DM, Smith JT, Vincent DE, et al (2007) Mouse Aurora A: expression in Escherichia coli and purification. Protein expression and purification 54: 139–146. 17434748 10.1016/j.pep.2007.03.002

[41] Liu Z-P, Galindo RL, Wasserman SA (1997) Phosphorylation of Cactus by CKII is required for wild-type Cactus function in Drosophila embryos. Genes & Development 11: 3413–3422. 9407033 10.1101/gad.11.24.3413 PMC316825

[42] Belvin MP, Jin Y, Anderson KV (1995) Cactus protein degradation mediates Drosophila dorsal-ventral signaling. Genes & Development 9: 783–793. 7705656 10.1101/gad.9.7.783

[43] Barroga CF, Stevenson JK, Schwarz EM, Verma IM (1995) Constitutive phosphorylation of I kappa B alpha by casein kinase II. Proceedings of the National Academy of Sciences of the United States of America 92: 7637–7641. 7644469 10.1073/pnas.92.17.7637 PMC41200

[44] Roth S, Hiromi Y, Godt D, Nüsslein-Volhard C (1991) Cactus, a maternal gene required for proper formation of the dorsoventral morphogen gradient in Drosophila embryos. Development 112: 371–388. 1794309 10.1242/dev.112.2.371

[45] Whalen AM, Steward R (1993) Dissociation of the dorsal-cactus complex and phosphorylation of the dorsal protein correlate with the nuclear localization of dorsal. Journal of Cell Biology 123: 523–534. 8227123 10.1083/jcb.123.3.523 PMC2200115

[46] Gillespie SK, Wasserman SA (1994) Dorsal, a Drosophila Rel-like protein, is phosphorylated upon activation of the transmembrane protein Toll. Molecular & Cellular Biology 14: 3559–3568. 8196601 10.1128/mcb.14.6.3559-3568.1994 PMC358723

[47] Drier EA, Govind S, Steward R (2000) Cactus-independent regulation of Dorsal nuclear import by the ventral signal. Current Biology 10: 23–26. 10660298 10.1016/s0960-9822(99)00267-5

[48] Edwards DN, Towb P, Wasserman SA (1997) An activity-dependent network of interactions links the rel protein Dorsal with its cytoplasmic regulators. Development 124: 3855–3864. 9367441 10.1242/dev.124.19.3855

[49] Kidd S (1992) Characterization of the Drosophila cactus locus and analysis of interactions between cactus and dorsal proteins. Cell 71: 623–635. 1423619 10.1016/0092-8674(92)90596-5

[50] Yang J, Steward R (1997) A multimeric complex and the nuclear targeting of the Drosophila Rel protein Dorsal. Proc Natl Acad Sci U S A 94: 14524–14529. 9405646 10.1073/pnas.94.26.14524 PMC25042

[51] Xiao T, Towb P, Wasserman SA, Sprang SR (1999) Three-dimensional structure of a complex between the death domains of Pelle and Tube. Cell 99: 545–555. 10589682 10.1016/s0092-8674(00)81542-1 PMC4372121

[52] Letsou A, Alexander S, Wasserman SA (1993) Domain mapping of tube, a protein essential for dorsoventral patterning of the Drosophila embryo. EMBO Journal 12: 3449–3458. 8253071 10.1002/j.1460-2075.1993.tb06019.x PMC413621

[53] Galindo RL, Edwards DN, Gillespie SK, Wasserman SA (1995) Interaction of the pelle kinase with the membrane-associated protein tube is required for transduction of the dorsoventral signal in Drosophila embryos. Development 121: 2209–2218. 7635064 10.1242/dev.121.7.2209

[54] Ho SN, Hunt HD, Horton RM, Pullen JK, Pease LR (1989) Site-directed mutagenesis by overlap extension using the polymerase chain reaction. Gene 77: 51–59. 2744487 10.1016/0378-1119(89)90358-2

